# Effects of biochar-based controlled release nitrogen fertilizer on nitrogen-use efficiency of oilseed rape (*Brassica napus* L.)

**DOI:** 10.1038/s41598-020-67528-y

**Published:** 2020-07-06

**Authors:** Jiayuan Liao, Xiangrong Liu, Ang Hu, Haixing Song, Xiuzhi Chen, Zhenhua Zhang

**Affiliations:** 1 Southern Regional Collaborative Innovation Center for Grain and Oil Crops in China, College of Resources and Environmental Sciences, Hunan Agricultural University, Changsha, 410128 China; 2 Guangdong Province Key Laboratory for Climate Change and Natural Disaster Studies, School of Atmospheric Sciences, Sun Yat-Sen University, Guangzhou, 510275 China; 3 Hengyang Branch of Hunan Tobacco Company, Hengyang, 421600 China; 4 National Engineering Laboratory On Soil and Fertilizer Resources Efficient Utilization, Changsha, 410128 China; 5 Hunan Provincial Key Laboratory of Farmland Pollution Control and Agricultural Resources Use, Hunan Provincial Key Laboratory of Nutrition in Common University, Changsha, China

**Keywords:** Biochemistry, Element cycles

## Abstract

Biochar-based controlled release nitrogen fertilizers (BCRNFs) have received increasing attention due to their ability to improve nitrogen-use efficiency (NUE) and increase crop yields. We previously developed a novel BCRNF, but its effects on soil microbes, NUE, and crop yields have not been reported. Therefore, we designed a pot experiment with five randomised treatments: CK (without urea and biochar), B (addition biochar without urea), B + U (biochar mixed urea), Urea (addition urea without biochar), and BCRNF (addition BCRNF), to investigate the effects of BCRNF on nitrifiers and denitrifiers, and how these impact nitrogen supply and NUE. Results of high-throughput sequencing revealed bacterial community groups with higher nutrient metabolic cycling ability under BCRNF treatment during harvest stage. Compared to Urea treatment, BCRNF treatment stimulated nitrification by increasing the copy number of the bacterial *amoA* gene and reducing nitrous oxide emission by limiting the abundance of *nirS* and *nirK*. Eventually, BCRNF successfully enhanced the yield (~ 16.6%) and NUE (~ 58.79%) of rape by slowly releasing N and modulating the abundance of functional microbes through increased soil nitrification and reduced denitrification, as compared with Urea treatment. BCRNF significantly improved soil NO_3_^−^, leading to an increase in N uptake by rape and NUE, thereby promoting rape growth and increasing grain yield.

## Introduction

Nitrogen is often the limiting factor for plant growth, thereby making plant N availability a critical factor for primary productivity in terrestrial ecosystems^1^. Nitrogen fertilization is an important agricultural practice for crop growth and is critical for sustaining global crop yields. However, the excessive use of N fertilizers in agroecosystems negatively affects the environment through nitrate leaching, runoff, and volatilization as the greenhouse gas nitrous oxide is released into the atmosphere. This results in low N-use efficiency (NUE) and low net economic returns^2–5^. Therefore, the enhancement of NUE in agricultural ecosystems is essential to address challenges in food security, environmental degradation, and climate change^6^.

Biochar is a porous carbonaceous solid produced by heating various biomass feedstocks under high temperatures in an oxygen-limited environment. Biochar has the potential to improve N recycling in agricultural soil–plant systems^7^. The application of biochar to agricultural soils reduces N_2_O emissions^8^, decreases N leaching^9^, improves soil nitrogen availability, increases crop productivity, and promotes the activity of soil microbes^10^. Biochar strongly adsorbs various nutrient ions, such as ammonium, nitrate, phosphate, and potassium through its numerous functional groups, such as carboxylic, hydroxyl, lactone, chromene, and ketone groups, resulting in the loading of nutrients and reduced soil N loss^11–14^. Recent studies have shown that biochar-based fertilizers significantly increase the productivities of rice, cabbage, and green pepper, while enhancing total NUE^15–18^. Moreover, biochar alone or co-applied with fertilizer promotes halophyte growth in coastal soil, resulting in improved soil health, enhanced nutrient availability, and elevated bacterial activity and abundance related to nutrient transformations^19^. For instance, a study has shown that biochar-based fertilizer amendment improves karst soil nutrient conditions; increases the microbial biomasses of C, N, and P; induces microbial community structural shifts; and increases bacterial community network complexity^20^. Most studies have focused on the mechanisms of biochar-based fertilizers. However, the effects of biochar-based, slow-release fertilizers on soil bacterial communities and how these drive soil N availability to impact plant physiology and NUE remain unknown.

Microorganisms play a key role in the N cycle^21^. Biochar amendment affects soil microbial abundance, activity, and community composition because of changes in soil environmental conditions^7,22^. The genes involved in N cycling include these marker genes: *nifH* (encodes nitrogenase, a key enzyme for N fixation), *amoA* (encodes ammonia monooxygenase, a key enzyme for nitrification), *nirK* and *nirS* (encode nitrite reductases, key enzymes for denitrification), and *nosZ* (encodes nitrous oxide reductase, a key enzyme for denitrification). These genes are often used to assess the functional abundance and diversity of soil microbial communities^23,24^. Previous studies have shown that the abundance of ammonia-oxidising bacteria (AOB) is significantly increased by the application of cotton stalk biochar, suggesting its potential utility in the enhancement of nitrification^25^. Conversely, Wang et al. found a reduced abundance of AOB following the addition of peanut shell biochar to acidic soil^26^. Denitrification reduces soil NO_3_^−^–N availability and is affected by biochar application. Many studies have found that the abundance of *nirK*, *nirS*, and *nosZ* is affected by biochar application^27–29^. For instance, Harter et al. found that biochar application results in an increase in the relative gene and transcript copy numbers of bacterial *nosZ*^28^. However, most of these studies either overlooked the effects of plant–soil interactions or focused on isolated soil. Only a few studies comprehensively evaluated the effects of changes in nitrogen availability and plant–soil interactions on soil bacterial communities in agroecosystems, particularly on genes involved in N-cycling functions.

Rape (*Brassica napus* L.), an important economic crop in China, accounted for approximately 21.0% and 20.4% of the global cultivated produce in 2014 and 2015, respectively^30,31^. *B. napus* has a high N demand, and its biomass and seed production can be increased by improving soil N availability^32,33^. Previously, we hydrothermally synthesised a novel BCRNF to control the slow release of N using polyvinyl alcohol, bentonite, and urea-loaded biochar. We found that BCRNF dramatically improves N release compared to a urea and biochar–urea mixture, thereby making BCRNF potentially useful in sustainable and green agriculture and food security^34^. However, the effects of BRCNF on soil fertility, bacteria, plant growth, and NUE and its underlying mechanisms in agricultural ecosystems remain to be elucidated. Here, we performed a pot experiment in a greenhouse to study the impact of BCRNF application on the NUE of rape and reveal the underlying mechanisms of BCRNF-stimulated transformation in agricultural production. The aim of this research was to investigate (1) whether BCRNF application influences soil properties and N availability; (2) if true, how plant physiology, N uptake, and NUE of *B. napus* respond to BCRNF; and (3) how microorganisms and the abundance of functional genes associated with N cycling are related to N bioavailability in BCRNF-treated soils.

## Results

### Soil bacterial community composition

**Figure 1:**
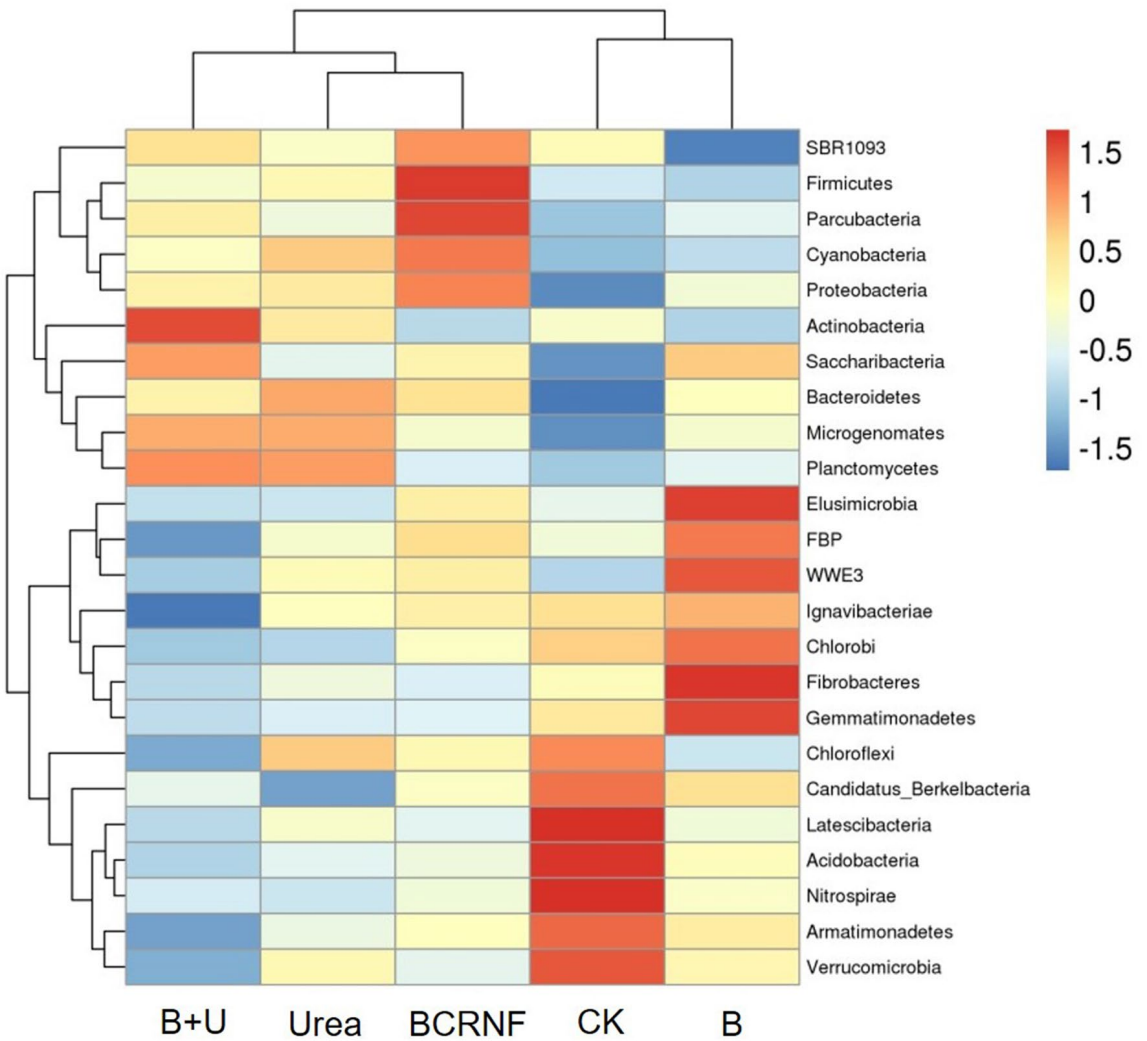
Distribution of the top 25 abundant bacteria at the phylum level and their cluster analysis under different treatments as visualised by heatmaps (variables clustering on the vertical axis). CK: untreated soil; B: soil treated with biochar; B + U: soil treated with biochar and urea; Urea: soil treated with only urea as nitrogen fertilizer; BCRNF: soil treated with BCRNF as nitrogen fertilizer. The colour intensity of the scale indicates the relative abundance of each phylum. Relative abundance is defined as the number of sequences affiliated with that taxon divided by the total number of sequences per sample (%).

The operational taxonomic unit (OTU) richness (estimated by ACE and Chao1 indices) of bacteria was impacted by the type of N fertilizers (Table S1). The bacterial α-diversity (Shannon and Simpson indices) of the soils subjected to the N-fertilizer treatments was significantly lower than that of the control. The most abundant phyla were Proteobacteria, Actinobacteria, Gemmatimonadetes, Acidobacteria, Chloroflexi, Saccharibacteria, Bacteroidetes, Firmicutes, Verrucomicrobia, and Armatimonadetes, accounting for more than 91% of the bacterial sequences in all treated soil (Fig. S1). The relative abundance of Acidobacteria, Armatimonadetes, Latescibacteria, Nitrospirae, and Verrucomicrobia was lower in the soils with the N-fertilizer and biochar (B) treatments than in the soils from the CK group (Fig. 1). The abundance of Proteobacteria, Firmicutes, Cyanobacteria, Parcubacteria, Saccharibacteria, and SBR1093 was higher and the abundance of Bacteroidetes, Actinobacteria, Microgenomates, Planctomycetes, and Chloroflexi was lower with BCRNF addition than with Urea treatment.

To further compare the impact of urea and biochar controlled-release N fertilizer on the bacterial community composition, bacteria with a relative abundance of > 1.5% under the Urea and BCRNF treatments were used for further analysis at the family level. The relative abundance of Acidothermaceae, Sphingomonadaceae, uncultured-bacterium-o-JG30-KF-AS9, Hyphomicrobiaceae, Burkholderiaceae, Xanthomonadaceae, and ODP1230B8.23 increased under the BCRNF treatment (Fig. S2). Furthermore, the relative abundance of Sphingobacteriaceae, DA101-soil-group, Intrasporangiaceae, Planctomycetaceae, Bradyrhizobiaceae, Oxalobacteraceae, Bacillaceae, and Erythrobacteraceae increased under the Urea treatment.

**Figure 2:**
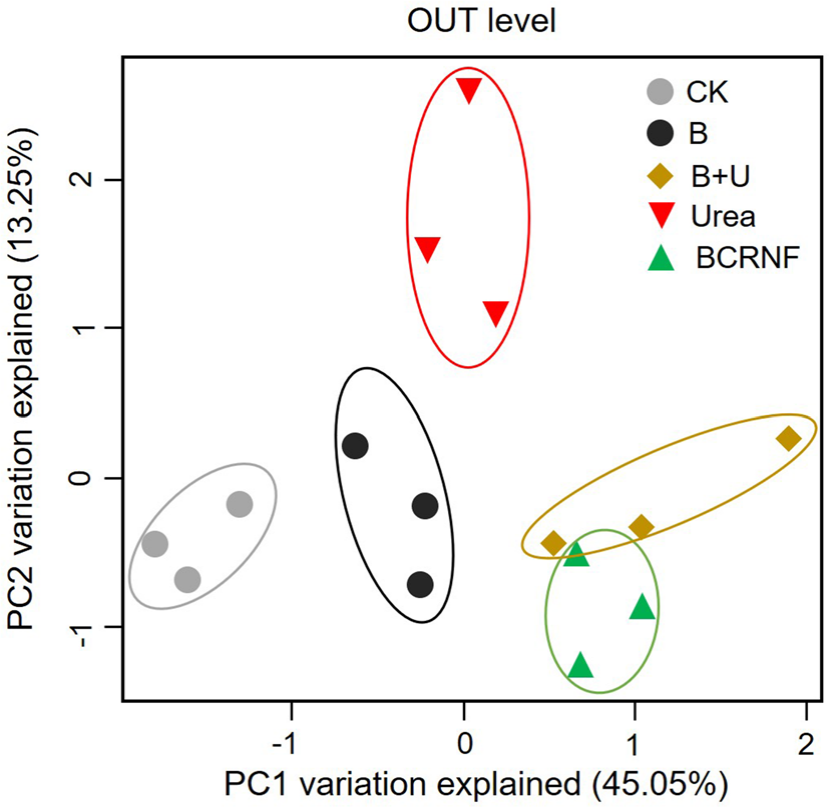
Principal component analysis (PCA) to compare bacterial composition. The percentages on the axes are the percent of variance explained by the first (PCA1) and second (PCA2) principal components under different treatments. CK: untreated soil; B: soil treated with biochar; B + U: soil treated with biochar and urea; Urea: soil treated with only urea as nitrogen fertilizer; BCRNF: soil treated with BCRNF as nitrogen fertilizer.

Principal component analysis (PCA) of the bacterial community structure was used to explore the variations in bacterial community composition among treatments (Fig. 2). The first axis of the PCA (PCA1) contributed 45.05% of the variation in the OTUs, whereas PCA2 accounted for 13.25% of the variation. The cumulative contribution rate was 58.30%. The bacterial community with CK treatment was separated from that with B, Urea, B + U, and BCRNF treatments by PCA1. The bacterial communities with urea and BCRNF treatments were also separated by PCA1. However, PCA1 and PCA2 did not separate the bacterial community composition between BCRNF and B + U treatments.

### Soil enzyme activities and microbial biomass

Soil enzyme activities, namely urease and fluorescein diacetate (FDA) hydrolysis, were increased by biochar treatments, and no differences among B + U and BCRNF treatments were observed (Fig. S3). BCRNF treatment led to a significantly higher urease activity than did Urea treatment for soil sampled during the harvest stages (Fig. S3a). Soil FDA activity (hydrolytic activity) was also strongly influenced by various treatments (Fig. S3b). Except when compared to B + U treatment, the activity under B treatment was dramatically higher than that under the other treatments in all stages. There were no differences in FDA activity under Urea and BCRNF treatments during the seedling stage. However, a significant decrease under Urea treatment was observed during the harvest stage.

Different treatments affected the microbial biomass (Fig. S4). Soil microbial biomass carbon (SMBC) increased significantly under Urea treatment and was higher under Urea treatment than under all other treatments during the seedling stage, whereas a significant increase in SMBC was observed under BCRNF treatment when compared to that under Urea treatment during the harvest stage (Fig. S4a). All biochar-containing treatments exhibited significantly decreased soil microbial biomass N (SMBN) compared to that of the CK group and Urea treatments during the seedling stage, but no significant difference during the harvest stage was observed (Fig. S4b). The soil microbial biomass P (SMBP) was not significantly different between the Urea and BCRNF treatments during the seedling stage, but the SMBP under BCRNF treatments was significantly greater than that under Urea treatments during the harvest stage (Fig. S4c).

### Nitrification and denitrification

The nitrogen and biochar treatments affected the potential nitrification rates in the soil (Fig. S5). No differences were observed under the various treatments during the seedling and bolting stages, but nitrification rates were significantly increased in the nitrogen and biochar-treated soils during the flowering and harvest stages. The nitrification rates under the BCRNF treatment were significantly greater than those under the Urea and B + U treatments during the flowering and harvest stages.

**Figure 3:**
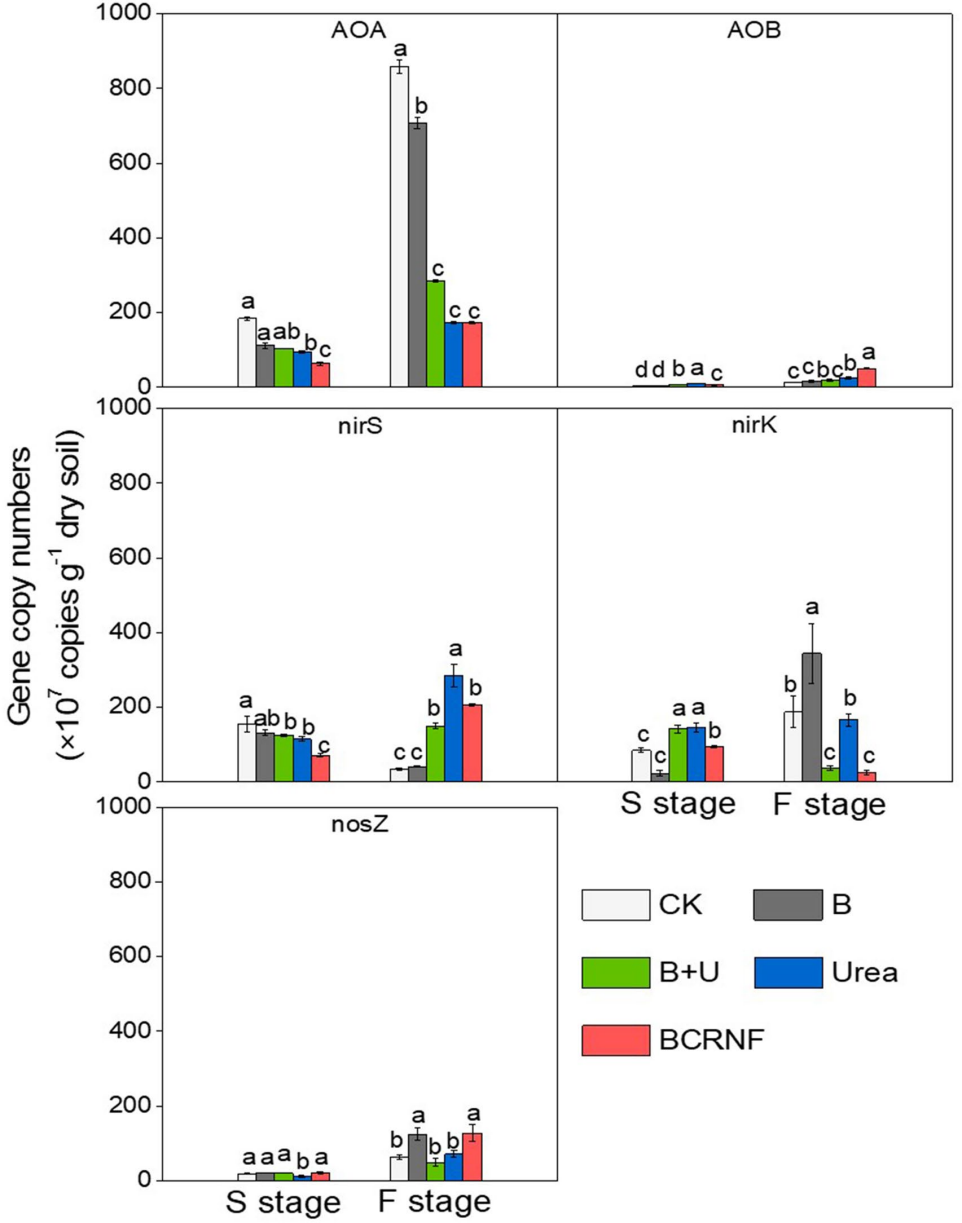
Soil functional gene abundance level under different treatments. S stage: seedling stage; F stage: flowering stage. CK: untreated soil; B: soil treated with biochar; B + U: soil treated with biochar and urea; Urea: soil treated with only urea as nitrogen fertilizer; BCRNF: soil treated with BCRNF as nitrogen fertilizer.

Real-time PCR assays of gene copy numbers in nitrifiers and denitrifiers were used to assess nitrification and denitrification, respectively (Fig. 3, Table S2). The AOA abundance in soils under the Urea, B + U, and BCRNF treatments significantly decreased during the seedling and flowering stages. In contrast, the abundance of AOB significantly increased with N fertilizer treatments compared to that in CK and B soils, whereas the abundance of AOB under BCRNF treatment was significantly higher than that under Urea treatment during the seedling and flowering stages. Regarding denitrifiers, the abundance of *nirS* and *nirK* under BCRNF treatment was significantly decreased compared to that under Urea treatment during the seedling and flowering stages. The abundance of *nosZ* in the BCRNF-treated soil significantly increased compared to that in the Urea-treated soil during the flowering stage.

**Figure 4:**
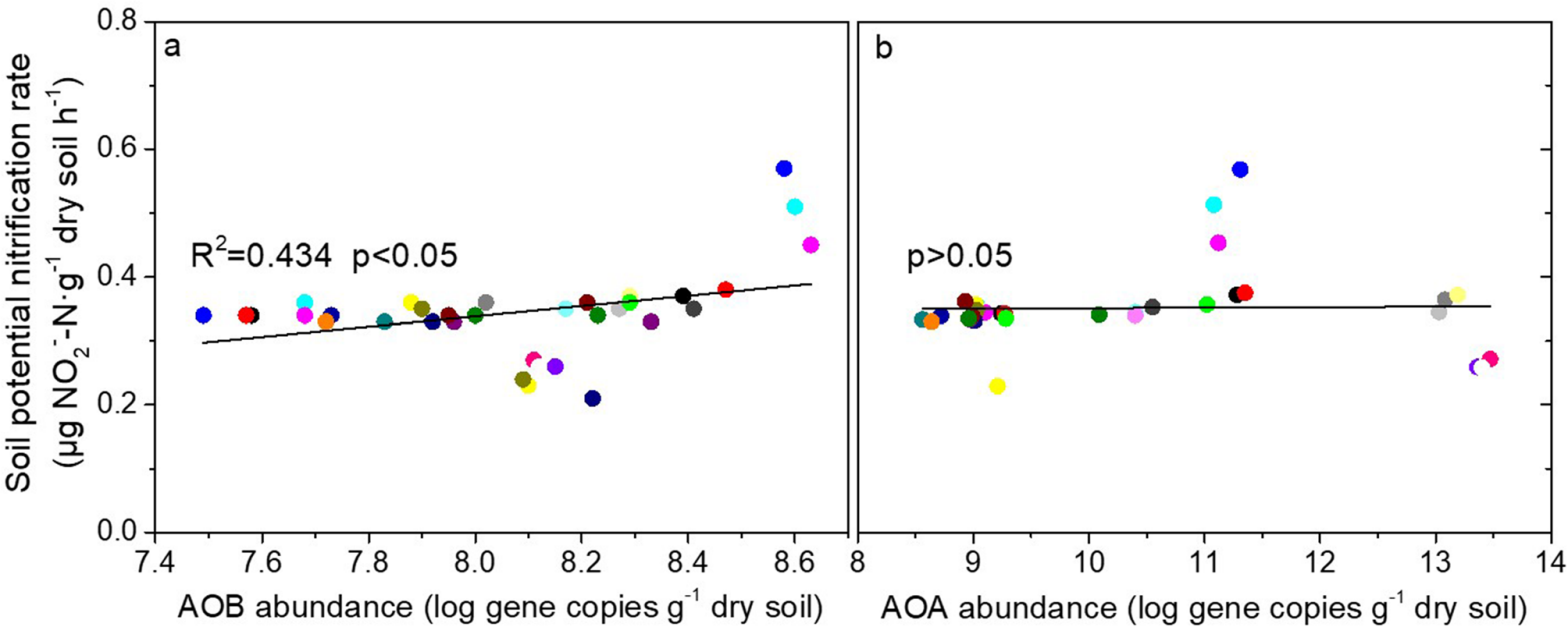
The relationships (**a**) between nitrification potential and ammonia-oxidising bacteria (AOB) *amoA* copy numbers and (**b**) between nitrification potential and ammonia-oxidising archaea (AOA) *amoA* copy numbers as determined by regression analysis. Gene copy numbers were log transformed before regression analysis.

Regression analysis indicated a dramatic difference in potential nitrification rates and AOA and AOB abundance in soil (Fig. 4). The AOB abundance was exponentially and positively correlated with soil nitrification potential (R^2^ = 0.434, *p* < 0.05). However, no positive or negative correlation in potential nitrification rates and AOA abundance (*p* > 0.05) was revealed.

### Soil inorganic N and other properties

Water incubation experiments were used to characterize the nutrient release rate and cumulative amount of released nutrients of BCRNF (Fig. S6). Urea exhibited a quick release rate with almost 100% of the nitrogen released to the water after 3 days. However, the cumulative release rate of N into water with BCRNF only reached 74.3% after 28 days. The nutrient release plots show the control release behaviour of BCRNF.

**Figure 5:**
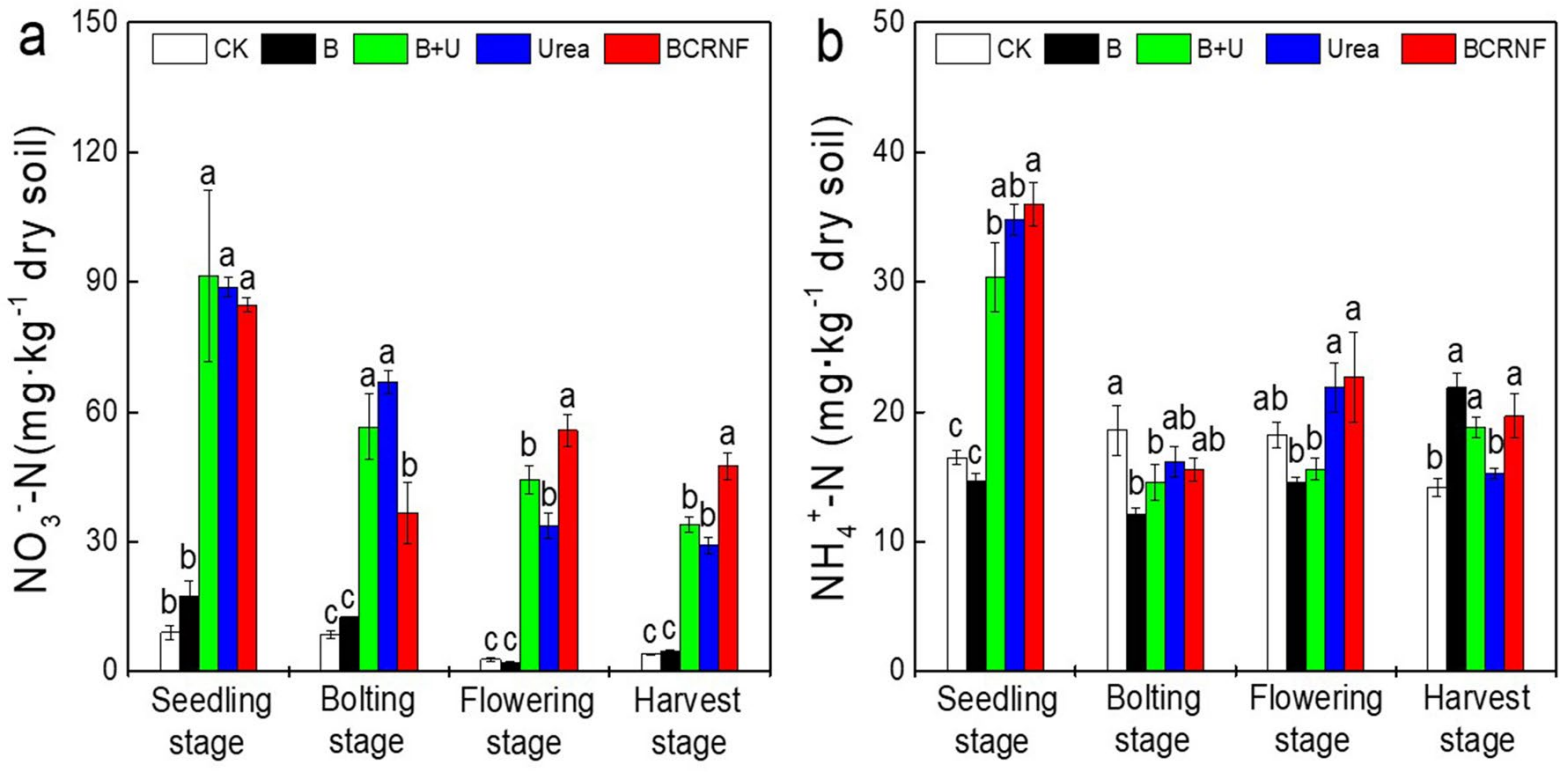
Effect of fertilizer treatments on nitrate nitrogen (**a**), and ammonium nitrogen (**b**) of soil. CK: untreated soil; B: soil treated with biochar; B + U: soil treated with biochar and urea; Urea: soil treated only with urea as nitrogen fertilizer; BCRNF: soil treated with BCRNF as nitrogen fertilizer. Different small letters indicate significant differences between different treatments (*p* < 0.05).

The different treatments affected nitrate N and ammonium N concentrations in the soils (Fig. 5). Nitrogen fertilizer treatments (B + U, Urea, and BCRNF) increased the NO_3_^−^–N concentration during the seedling, bolting, and flowering stages more than the CK and B treatments did, whereas no differences between CK and B treatments were observed (Fig. 5a). During the seedling stage, the NO_3_^−^–N concentration under BCRNF treatment was not significantly different from that under Urea and B + U treatments. The NO_3_^−^–N concentrations under Urea and B + U treatments were not significantly different during the bolting stage, but both were significantly higher than that with BCRNF treatment. However, during the reproductive growth stage (flowering and harvest stages), the NO_3_^−^–N concentration of soils under BCRNF treatment was the highest among all samples. The NH_4_^+^ − N concentration increased during the seedling stage under the N fertilizer treatments, and no differences between the Urea and BCRNF treatments during the bolting and flowering stages were observed (Fig. 5b). During the harvest stage, there were no differences among the B, B + U, and BCRNF treatments, but all significantly increased the NH_4_^+^–N concentration over that observed with Urea treatment.

The addition of BCRNF influenced the physical and chemical properties of the soil (Table S3). At the seedling stage, soil pH significantly decreased with B + U and BCRNF treatments compared to that with Urea treatment, but no significant difference was found among the CK, B, and Urea treatments. During the harvest stage, nitrogen fertilizer treatments significantly reduced soil pH, and there was no difference between the CK and B treatments. During the seedling and harvest stages, B and BCRNF treatments significantly increased soil organic matter (SOM) content. The Urea treatment increased the value of total nitrogen (TN), whereas under BCRNF treatment, no significant difference was found from the no-N control during the seedling stage. The concentration of total K (TK) and available potassium (AK) was not significantly different between the BCRNF and Urea treatments in the seedling and harvest stages. However, BCRNF treatment in the harvest stage increased the values of TN, total P (TP), and Olsen-P compared to those observed with Urea treatment.

### Plant N bioavailability and other nutrients

**Figure 6:**
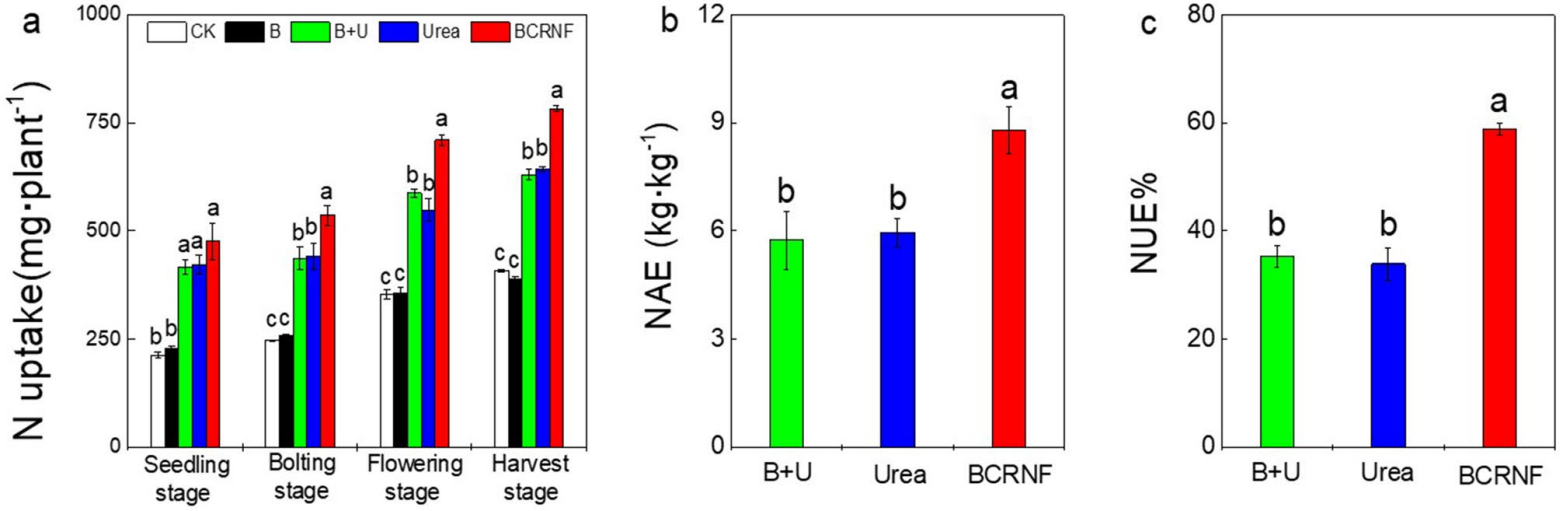
Effect of fertilizer treatments on nitrogen uptake (**a**), N agronomic efficiency (**b**), and nitrogen use efficiency (**c**) of *Brassica napus*. CK: untreated soil; B: soil treated with biochar; B + U: soil treated with biochar and urea; Urea: soil treated with only urea as nitrogen fertilizer; BCRNF: soil treated with BCRNF as nitrogen fertilizer. Different small letters indicate significant differences between different treatments (*p* < 0.05).

The application of N fertilizer significantly impacted the uptake and accumulation of N by *B. napus*, but no significant differences between the CK and B treatments were found in all stages (Fig. 6a). N uptake was higher during the bolting, flowering, and harvest stages with BCRNF treatment than with B + U or Urea treatment, and no significant difference was observed between the B + U and Urea treatments in all stages. Furthermore, the result by ^15^N tracer technique showed that more ^15^N from BCRNF uptake by rape during the bolting, flowering, and harvest stages, and more ^15^N was distributed to the grain in harvest stage under BCRNF treatment (Fig. S8). Nitrogen agronomic efficiency (NAE) significantly increased under BCRNF treatment (Fig. 6b). The BCRNF treatment had the highest NAE among all treatments, with a ~ 27.7% higher NAE than the Urea treatment. The BCRNF treatment also had a significantly increased NUE (~ 58.79%) compared to that of other treatments. However, no obvious differences in NAE and NUE were observed between the B + U and Urea treatments (Fig. 6c). Activities of the N-assimilatory enzymes nitrate reductase (NR) and glutamine synthetase (GS), root activity, and N physiological use efficiency (NPUE) of *B. napus* were also assessed (Fig. S7). However, the enzymatic activities and NPUE were not significantly different between the BCRNF and Urea treatments in all stages.

Different treatments altered plant nutrients (Table S4). Plant TN concentration increased significantly with the nitrogen fertilizer compared to that of the control during the seedling, flowering, and harvest stages, but this was not significantly different among the B + U, Urea, and BCRNF treatments. BCRNF treatment increased the TP concentration of the plant more than Urea treatment in the seedling and harvest stages. During the flowering stage, the TP concentration under the BCRNF treatment was not different than that under the B + U and Urea treatments. There were no differences in the TK concentration with the Urea and BCRNF treatments during the seedling, bolting, and flowering stages. During the harvest stage, BCRNF treatment increased the TP and TK concentrations more than the CK and Urea treatments did.

### Plant biomass and grain yield

**Figure 7:**
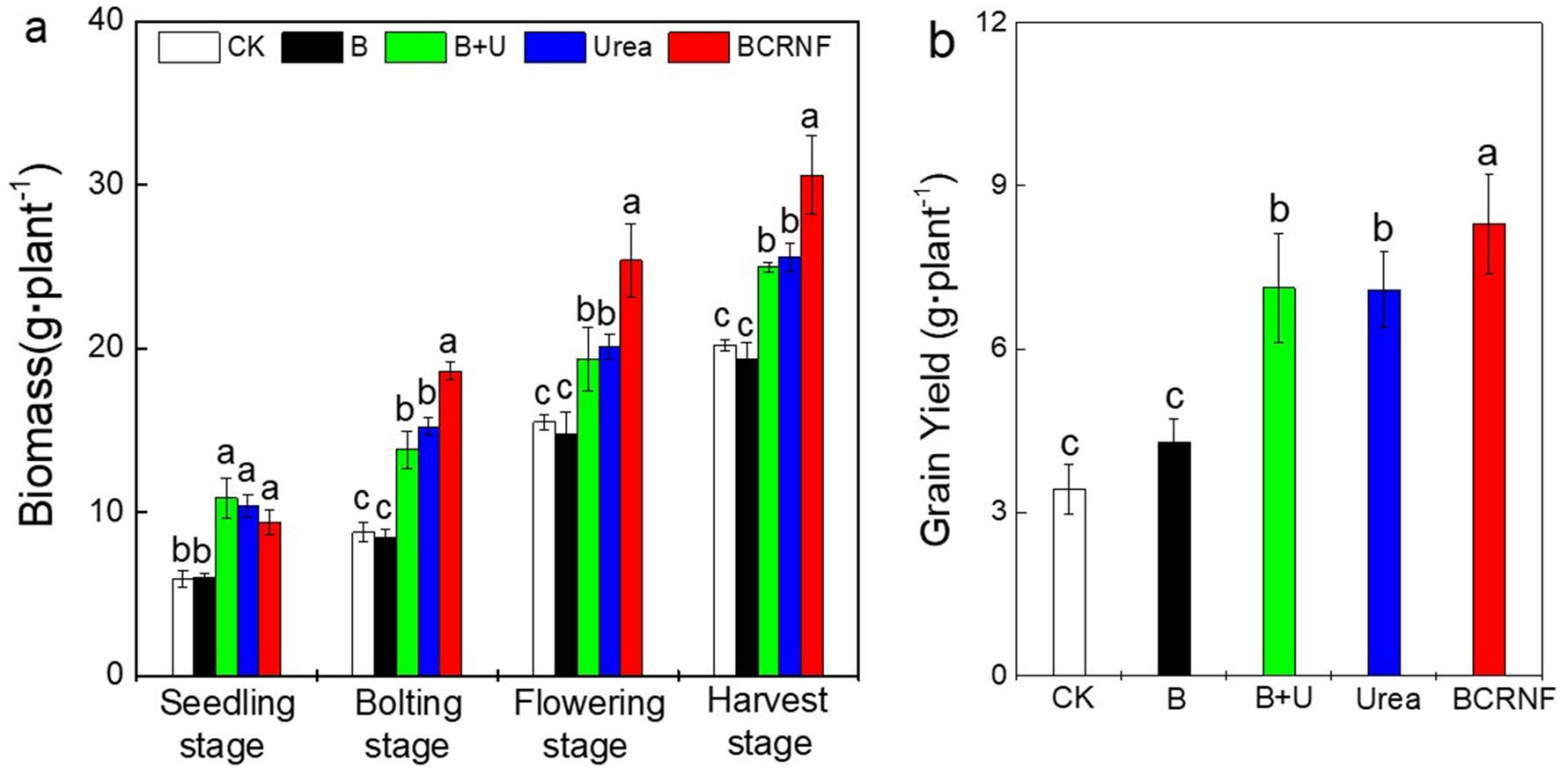
Effect of fertilizer treatments on dry weight of biomass (**a**) and grain yield (**b**) of *Brassica napus*. CK: untreated soil; B: soil treated with biochar; B + U: soil treated with biochar and urea; Urea: soil treated with only urea as nitrogen fertilizer; BCRNF: soil treated with BCRNF as nitrogen fertilizer. Different small letters indicate significant differences between different treatments (*p* < 0.05).

The accumulation of dry weight biomass of *B. napus* increased more under the nitrogen fertilizer treatments than in the control during the four planting stages (Fig. 7a). The biomass was not significantly different among the B + U, Urea, and BCRNF treatments during the seedling stage. However, biomass accumulation of plants under the BCRNF treatment was significantly greater than that under the Urea and B + U treatments during the bolting, flowering, and harvest stages. The biomass accumulation under the Urea treatment was similar to B + U treatment during the four stages.

Grain yield was impacted by the application of N fertilizers (Fig. 7b). All urea-containing treatments produced significantly higher yields than the CK group and biochar treatments. The grain yield of plants under the BCRNF treatment was significantly higher than that under the other treatments. However, the yield of the plants under the B + U treatment was approximately the same as that under the Urea treatment, and BCRNF yielded ~ 16.6% more grain than Urea did.

## Discussion

N is often the limiting factor for plant growth, thereby making N availability critical for primary productivity in terrestrial ecosystems^1^. In accordance with this study, we found a significantly higher NO_3_^−^–N concentration in BCRNF-treated soil than in the other treated soils during the reproductive growth period of *B. napus* and a dramatically improved N uptake (Figs. 5, 6). A previous study has demonstrated that controlled release nitrogen fertilizer (CRNF) continuously supplies nitrogen during the late crop growth period through its slow release, thereby significantly increasing crop yield^35^. In the present study, similar results were achieved, and there are several possible reasons for this. First, urea had a rapid release rate with almost 100% of the nitrogen released into the water after 3 days. However, N release from BCRNF was lower than that from Urea during initial stages (Fig. S6). The N release profiles of BCRNF followed analogous parabolic diffusion models, indicating that release is a combined process of dissolution, adsorption, and diffusion^36^. Our previous research found that urea particles filled the inner pores and channels of biochar, and a new organic complex was generated. X-ray diffraction, Fourier transform infrared spectroscopy, and X-ray photoelectron spectroscopy demonstrated the favourable controlled-release properties of BCRNF^34^. This property of BCRNF ensures the supply of N nutrients during different growth stages. Second, BCRNF changed soil microbial community composition, increased soil enzyme activities, and increased N nitrification; thus, it improved N availability to the crop. These findings show that BCRNF ensures the supply of available N fertilizer during the late growth stages of rape to support growth, thereby promoting N uptake.

N uptake by plants was significantly higher under BCRNF treatment than under Urea treatment with the same N-application rate, resulting in significantly increased NUE and NAE after BCRNF addition (Fig. 6a–c). Total N uptake, NUE, and NAE under Urea treatment corresponded with those of B + U treatment, suggesting that small doses (< 1 t ha^−1^) of biochar mixed with urea does not promote plant growth. Studies revealed that in the middle and late stages of rape development, N accumulation can delay the senescence of vegetative organs and accelerate the transport of nitrogen to the rapeseed and increase grain yield^37,38^. The ^15^N tracer technique has been widely used to determine the effects of fertilization practice and the uptake of N fertilizer use on *B. napus* yield in the plant–soil system. Our findings further indicated that N uptake had improved, and more N was distributed to the grain under BCRNF treatment (Fig. S8). The physiology of the plant affects the NUE; however, BCRNF had no obvious influence on NPUE (Fig. S7a), because the N-assimilating enzymes NR and GS of *B. napus* showed similar activities under both Urea and BCRNF treatments (Fig. S7c,d). These results suggest that nitrogen metabolism does not cause differences in NUE with Urea and BCRNF treatments mainly because BCRNF prolongs N release and improves NUE. A similar study showed that BCRNF generally outperforms Urea in reducing N losses, increasing NUE (~ 46%), and stimulating plant growth^18^.

**Figure 8:**
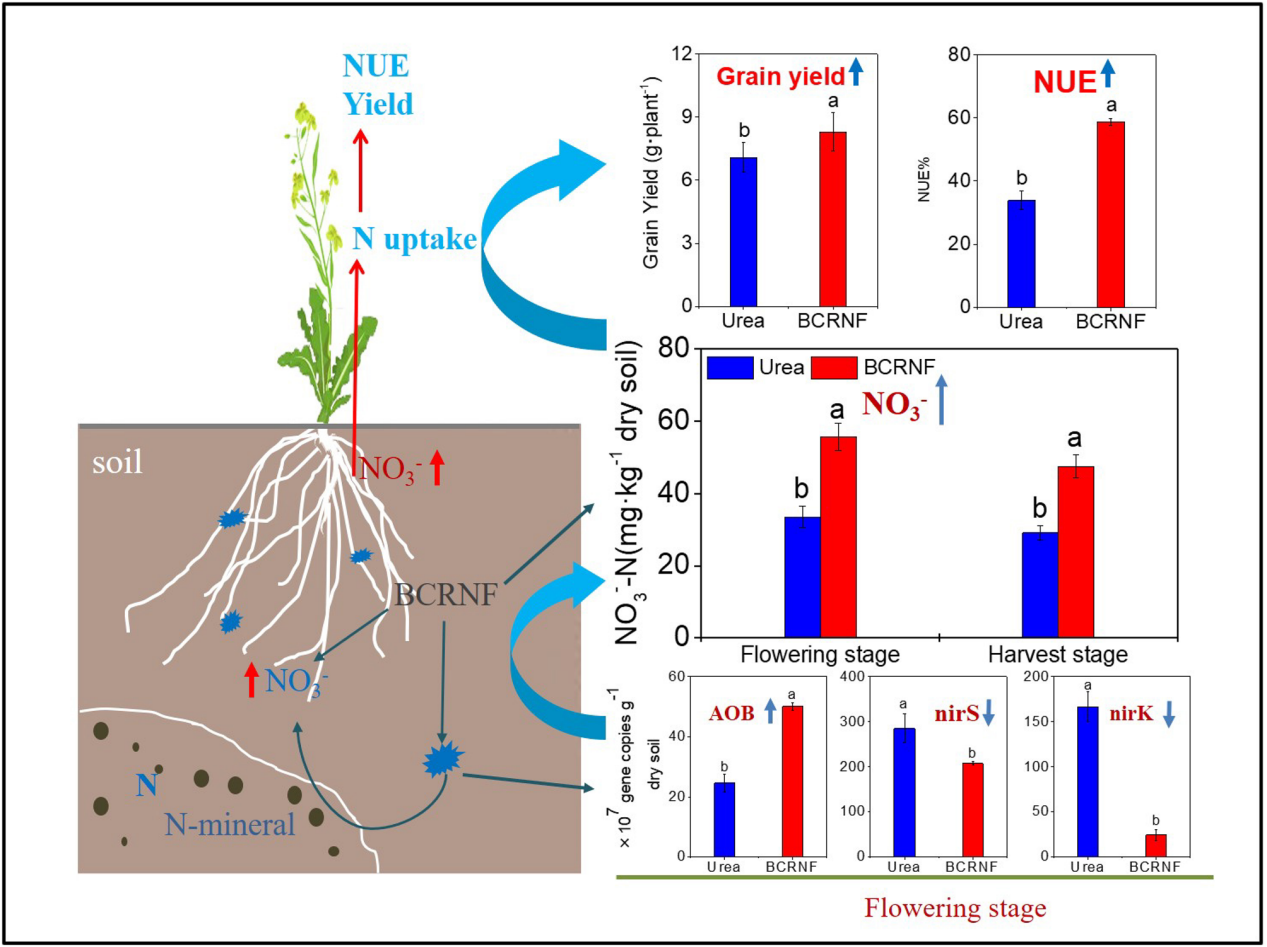
Conceptual diagram of significant treatment effects on soil and associated plant growth with BCRNF application.

Some studies have found that *B. napus* has a high N demand, and by improving soil N availability, its biomass and seed production can be increased^32,33^. To summarize, BCRNF mainly increases soil NO_3_^−^–N during the later stages of growth, thereby increasing N uptake of rape and NUE, eventually promoting rape growth and increasing grain yield. These findings indicate that BCRNF can be used to improve the biomass of rape relative to urea-only treatments. In this study, BCRNF enhanced NUE by increasing soil NO_3_^−^–N mainly through: (1) the slow release of N, thereby increasing soil NO_3_^−^–N; and (2) increased soil nitrification via AOB activity and reduced denitrification during later stages, thereby increasing soil NO_3_^−^–N for rape growth (Fig. 8).

The N cycle, driven by microbiological processes, is a complex biogeochemical cycle^39^. Microbial regulation influences the forms of N available for plant uptake, e.g., nitrification and denitrification^21,40^. However, there is no direct evidence of the mechanism by which biochar-based slow-release fertilizers regulate N cycling and subsequent N availability related to plant uptake. Nitrification is a key process involved in N cycling. Soil potential nitrification rates represent the ability of soil-nitrifying microbes to transform NH_4_^+^ to NO_3_^−^ and are affected by the abundance of nitrifying populations^41^. In this study, higher soil potential nitrification rates were observed with BCRNF treatment than with other treatments. The abundance of AOB was dramatically increased by the BCRNF treatment compared with that by the Urea treatment during the flowering stage, and soil potential nitrification rates were positively correlated with AOB, but not with AOA, abundance (Figs. 3, 4). This may explain the higher NO_3_^−^–N concentration observed in BCRNF soils, which occurred from the transformation of higher amounts of NH_4_^+^ to NO_3_^−^ due to the nitrifying microbes in the soil. AOB may also play an important role in the improved nitrification potential after BCRNF addition as observed in this study. Previous research has indicated that AOA controls nitrification under oligotrophic conditions without external N amendments and AOB is sensitive to fertilizer supply^42^. High N input increases AOB abundance^43^; therefore, higher soil N concentration may be a contributing factor in the higher AOB abundance in BCRNF-treated soil. Besides soil N content, higher C input has been reported to stimulate organic matter mineralization and enhance AOB growth^44^. Our finding showing that BCRNF significantly increases soil SOC (Table S3) supports the findings of Simonin et al. Therefore, BCRNF regulation of AOB abundance during the flowering and harvest stages is a key microbial mechanism to increase soil NO_3_^−^–N (Fig. 6).

Soil NO_3_^−^–N concentration is also affected by other microbes through denitrification. Reduced denitrification may also result in a higher NO_3_^−^–N concentration; therefore, it was speculated that factors other than higher soil potential nitrification rates lead to high NO_3_^−^–N concentrations in BCRNF-treated soil. In the present study, both *nirS* and *nirK* abundance was significantly reduced by BCRNF addition, which perhaps explains the higher NO_3_^−^–N concentration in BCRNF-treated soil. We also found that the abundance of *nosZ* dramatically improved in BCRNF-treated soil (Fig. 3). An enhanced copy number of *nosZ* has been reported to reduce conversion of N_2_O to N_2_, thereby potentially reducing greenhouse gas emissions^28^. Thus, our findings indicate that BCRNF may have enhanced environmental benefits versus traditional urea fertilizers. Nonetheless, the inherent mechanism remains to be investigated.

Previous studies have reported contrasting results about the effect of biochar on microbial community composition in soil^45^. However, no previous study has reported how BCRNF impacts soil microbes. FDA activity (hydrolytic activity) estimates the activities of microbes, which were significantly increased with biochar amendments compared to those with Urea treatment (Fig. S3). This indicates that biochar combined with urea stimulates microbial activity more effectively than traditional urea fertilizer. PCA analysis based on OTUs showed a dramatic change in bacterial community composition in the BCRNF-amended soil compared with that in soils treated with urea (Fig. 2). Canonical correlation analysis (CCA) of the bacterial communities obtained using family abundance indicated that SOM, TP, NH_4_^+^, and NO_3_^-^ were positively correlated with the bacterial community in BCRNF soil (Fig. S9), which was consistent with previous studies that found that soil fertility factors affect the microbial community^46^. The change in bacterial community composition with BCRNF treatment may be chiefly due to altered soil physicochemical properties and improved substrate availability (of C, N, and P) that results from the altered C input from biochar^47,48^. This is consistent with our hypothesis that BCRNF increases microbial activity and alters bacterial community, thereby enhancing soil nutrient availability and benefiting plant growth.

Proteobacteria are also called ‘copiotrophic’ bacteria, and most live in nutrient-rich conditions^49^. Proteobacteria were dominant under different treatments and increased more with BCRNF treatment than with Urea treatment (Figs. 1 and S2). The results suggested that soil nutrient conditions are higher under BCRNF treatment than under Urea treatment. Cyanobacteria are reported to be capable of N fixation^21^. Saccharibacteria comprise diverse species and play an important role in the degradation of organic compounds in soil under aerobic and nitrate-reducing conditions^50^. Thus, the increased abundance of Cyanobacteria and Saccharibacteria after BCRNF addition indicates that BCRNF has the potential to provide soil nutrients. Our study showed higher contents of TN, TP, Olsen-P, and NO_3_^−^–N in the soils during the late stage after BCRNF addition (Table S3, Fig. 6). Chloroflexi can severely inhibit the growth of crops^51^. The abundance of Chloroflexi declined in BCRNF-treated soil, which suggests that BCRNF-regulating microorganisms are beneficial to rape growth. At the family level, Hyphomicrobiaceae and Sphingomonadaceae were promoted by BCRNF addition (Fig. S2). A previous study revealed that these families play a key role in N cycling and the degradation of refractory pollutants, organic matter, and aromatic compounds^52^. For instance, a species belonging to *Devosia* (Hyphomicrobiaceae) is able to fix and modulate N symbiotically with plants^52^. BCRNF can maintain a sufficient N source (ammonium, nitrate) for microbes due to its various nutrient ions (ammonium, nitrate) in numerous functional groups (carboxylic, hydroxyl, lactone, chromene, ketone), which act as electron shuttles for accepting and donating electrons. This may promote colonization by large numbers of microorganisms with N-related functional genes and drive a series of microbial processes^53–56^. Our results showed that BCRNF amendment changes the composition of soil bacterial communities, and increases the abundance of functional genes related to N metabolic in the soil, which may accelerate N cycling; thus, N availability to crops is increased. This putative mechanism may explain the increase in biomass observed in *B. napus*.

## Conclusions

In this study, for the first time, the effects of BCRNF on N availability and plant-soil interactions on soil bacterial communities in agroecosystems were comprehensively evaluated, particularly on genes involved in N-cycling functions. The maintenance of soil NO_3_^−^–N concentration for the growth of *B. napus* is the main factor underlying the elevated NUE of *B. napus* upon the addition of BCRNF, as its slow release of N affects microbes involved in nitrification and denitrification. We evaluated the underlying mechanisms and found that BCRNF improves microbial activity, shifts bacterial community composition toward groups with high nutrient metabolic cycling ability, and increases N nitrification by stimulating *amoA* expression in AOB. This improves the abundance of AOB and stimulates nitrification, accelerating the transformation of NH_4_^+^ to NO_3_^−^ and reducing NO_3_^−^ (gas) loss by limiting the abundance of *nirS* and *nirK*. These processes were key to increasing soil NO_3_^−^–N concentration with BCRNF treatment. Our results also indicate that N_2_O emissions may be reduced by increasing the abundance of *nosZ* reducers in BCRNF-treated soil. The application of BCRNF should be encouraged as a partial substitution for high-yield fertilization by split application to increase rape yields and NUE. This also has the potential to reduce N_2_O pollution, but this possibility should be investigated in further studies of N_2_O emissions from soil. The results of this study are based on pot experiments; thus, these processes will be further studied in a field experiment in the future, to evaluate the effects of BCRNF on NUE in different agriculture ecosystems.

## Materials and methods

### Soil sampling and BCRNF preparation

Topsoil samples (0–20 cm) were collected from a paddy field in Anren County, Hunan Province in southern China (~ 26°17′ N–26°50′ N, ~ 113°05′ E–113°36′ E)^57^, air-dried, ground until able to pass through a 2-mm sieve, and thoroughly homogenised. Table S5 shows the basic soil properties. The preparations of biochar and BCRNF are summarised in the Supplementary Information. The N release characteristic of BCRNF was compared with tha t of urea alone and the biochar–urea mixture we previously reported^34^.

### Water incubation experiment

To evaluate N release characteristics^58^, 1 g each of urea, biochar–urea mixture (2.47:1), and BCRNF was placed into dialysis membrane tubes (molecular weight cut-off = 12 − 14 kDa). The dialysis tubes were immersed in 100 mL distilled water and incubated at 20 °C. After 1, 3, 7, 14, 21, 28, and 42 days, 5.0 mL of solution was collected in each dialysis bag, and additional distilled water was added to maintain a volume of 100 mL. The total nitrogen (TN) concentration of the solutions was determined using the Kjeldahl method^59^.

### Plant material and experimental site

The oilseed rape (*B. napus*) cultivar used was Xiangyou15, which is widely used in the Yellow River Delta^30^. Pot experiments were carried out between October 2017 to April 2018 at the experimental greenhouse of the Resources and Environment Base of the Hunan Agricultural University (28.177°N, 113.087°E), Changsha, China. This region has a subtropical monsoonal climate with an annual average temperature of 17.2 °C and annual precipitation of 1,300–1,500 mm^30^.

### Pot experiment

Five treatments using a randomised complete block design were carried out: CK control, B control, and three different nitrogen fertilizer treatments with equivalent N (200 kg ha^−1^):

1. Soil without N fertilizer and biochar (CK),
2. Soil amended with 3.31 g of biochar (equivalent ~ 1 t ha^−1^) and without N (B),
3. Soil amended with 3.31 g of biochar and 1.34 g of urea (B + U),
4. Soil amended with 1.34 g of urea and without biochar (Urea),
5. Soil amended with 4.65 g of BCRNF as N fertilizer (BCRNF).

BCRNF and urea (^15^N-labelled fertilizers) were used as N fertilizers; calcium magnesium phosphate, P fertilizer; and potassium chloride, K fertilizer (P and K fertilizers were used in all treatments.). The fertilizers were applied at a standard application rate: N, P, and K at 0.63, 1.57, and 0.39 g per pot, respectively, and equivalent to 200, 60, and 75 kg ha^−1^, respectively). Air-dried soil (6.25 kg) was mixed with the fertilizer or biochar in each pot (20 cm diameter, 25 cm height), and rape seedlings were transplanted into the pots, with one plant per pot. To eliminate disturbance from seed-associated microbes, we used germinated, sterilised, dehulled rape seeds on a mannitol salt agar medium, and transplanted seedling to the pots as 20-day-old plants. The pots were maintained at ~ 50% of the maximum water holding capacities (WHC) with distilled water during the incubation in each treated soil. Each treatment was conducted in triplicate on four sampling dates, and the pots were randomly distributed in a greenhouse under natural light. Soil and *B. napus* plants were sampled during the seedling stage (7 weeks), bolting stage (11 weeks), flowering stage (15 weeks), and harvest stage (24 weeks) from the beginning of the experiment in order to measure plant physiological parameters and soil properties. Samples at the seedling and flowering stages were analysed using quantitative PCR and those at the harvest stage were analyzed using sequencing.

### Soil chemical properties

Soil pH was determined in a 1:5 (w:v) soil-to-water slurry using a pH-meter (AB150, Fisher Scientific, USA). SOM was measured using an oxidation method with potassium dichromate^60^. TN was measured by an automatic azotometer (KDN-102F, Qianjian Ltd., Shanghai, China). TP was determined using sodium hydroxide fusion and measured by colorimetric analysis. Olsen phosphorus values extracted with 0.5 M NaHCO_3_ were measured by a segmented continuous flow analyser (QuAAtro, Bran + Luebbe, Norderstedt, Germany). TK was determined using flame photometry after sodium hydroxide fusion, and the available K was extracted with NH_4_OAc and measured by flame photometry. NO_3_^−^–N and NH_4_^+^–sssN were extracted with 2 M KCl solution at a soil/water ratio of 1:5 at 25 °C and measured using a Smart Continuous Flow Analyzer (SmartChem200, Shenzhen, China). Potential nitrification rates were measured using the chlorate inhibition method^61^.

### Soil enzyme activities

Hydrolytic activity evaluated the total microbial activity in soil using the FDA method and was spectroscopically measured at 494 nm^62^. Soil urease activity was tested using the Solarbio kit (Beijing, China) according to the manufacturer’s instructions.

### Soil microbial biomass

SMBC, SMBN, and SMBP were measured by the chloroform fumigation extraction method^63^.

### Plant physiological parameters

The ^15^N content of the plant and grain were measured by a continuous-flow isotope ratio mass spectrometer coupled with a C–N elemental analyser (ANCA-MS; PDZ Europa). The activity of the root was determined by the triphenyltetrazolium chloride method^64^. NR activity was measured according to the method reported by Fan et al.^65^. GS activity was measured according to the method reported by Wang et al.^4^. N concentration was measured using a Foss Auto Analyzer Unit (Kjeldahl 8,400). TP and TK concentrations were determined using inductively coupled plasma mass spectrometry (MARS5, CEM, USA). Grain yields were determined at the harvest stage. The accumulation of N was determined from the sum of the total dry matter weight and N concentration in different plant parts.

### DNA extraction, PCR amplification, and high-throughput sequencing

Soil total DNA was extracted from 0.5 g of fresh soil using the PowerSoil DNA isolation kit (MO BIO Laboratories, Carlsbad, CA, USA) according to the manufacturer’s instructions. Each treatment was conducted in triplicate at the harvest stage, a total of 15 samples. The DNA was quantified by spectrophotometry (NanoDrop One, Thermo Scientific, Waltham, MA, USA) and its integrity verified using 1.0% agarose gel electrophoresis. The DNA extracts were stored at − 80 °C until use.

The V3/V4 regions of the 16S ribosomal RNA gene were amplified using PCR (initial denaturation at 98 °C for 2 min followed by 30 cycles at 98 °C for 30 s, 50 °C for 30 s, and 1 min at 72 °C with a final extension at 72 °C for 5 min.) using the primers 338F (5′-ACTCCTACGGGAGGCAGCA-3′) and 806R (5′-GGACTACHVGGGTWTCTAAT-3′), which target conserved sequences found in bacteria. PCR amplification was performed with a 50 µL reaction mixture containing 10 μL of 5 × FastPfu buffer, 2 μL of 2.5 mM dNTPs, 1.5 μL of each primer (10 μmol), 0.2 μL of Q5 High-Fidelity DNA Polymerase, and 40 ng of template DNA. The PCR products were extracted from the agarose gel following electrophoresis (1.8% agarose) and purified using a MinElute® PCR Purification Kit (Sangon Biotech, China). Finally, all PCR products were quantified by Quant-iT™ dsDNA High-Sensitivity Reagent (Thermo Fisher, Waltham, Massachusetts, USA) and pooled. High-throughput sequencing of the bacterial rRNA genes was performed on the purified, pooled samples using the Illumina HiSeq 2,500 platform (2 × 250 paired ends) at Biomarker Technologies Corporation, Beijing, China.

Raw FASTQ files were demultiplexed and quality-filtered using QIIME (version 1.17). OTUs, clustered with 97% similarity cut-off using UPARSE and chimeric sequences, were identified and removed using UCHIME. The taxonomy of each 16S rRNA gene sequence was assigned using an RDP classifier against the Silva (SSU115) 16S rRNA database using a confidence threshold of 70%^66,67^. PCA was used to visualize the Bray–Curtis dissimilarity matrices based on the OTU data. We used heat maps to display the abundance of species in the different samples using the “vegan” package in R (version 3.6)^68^.

### Real-time quantitative PCR

Quantitative polymerase chain reaction (qPCR) was performed to quantify the abundance of functional marker gene (*amoA*, *nirK*, *nirS*, *nifH*, and *nosZ*) copy numbers using the SsoAdvanced SYBR Green Supermix. Each sample was measured in triplicate using a CFXCONNECT Real-Time PCR Detection System (Bio-Rad Laboratories, Hercules, CA, USA) and gene-specific primers adopted from previous studies. The primer plasmid standards, thermal programs, and reaction mixtures for qPCR are summarised in Table S6. Standard plasmids were gel-purified using an OMEGA quick PCR purification kit, ligated into the PMD-19T vector (Takara Cloning® Kit), and transformed into competent DH5α *Escherichia coli* (Takara). The white positive clones were selected for plasmid DNA extraction using an OMEGA Plasmid Extraction Kit and used as functional gene standards. Standard curves were constructed with plasmids containing cloned gene fragments. Results with correlation coefficients and amplification efficiencies above 0.98% and 99%, respectively, were used for downstream analyses.

### Statistical analysis

The data were analysed using SPSS 19.0 (IBM) software. Analysis of variance (ANOVA) and Duncan’s multiple range tests were used to evaluate the differences between the means of the three replicates under different treatments with a P value of 0.05 indicating statistical significance. Figures were generated using Origin 9.0 (Origin Lab) software. The nitrogen-use efficiency (NUE), nitrogen agronomic efficiency (NAE), and nitrogen physiological use efficiency (NPUE) were calculated using the following formulas:

$${\text{NUE }}\left( {\text{\% }} \right) = \frac{{{\text{Total}}\;{\text{N}}\;{\text{uptake}}\;{\text{of}}\;{\text{plant}}\;{\text{from}}\;{\text{the}}\;{\text{treatment}} - {\text{Total }}\;{\text{N}}\;{\text{uptake}}\;{\text{from }}\;{\text{the }}\;{\text{control}}}}{{{\text{Total}}\;{\text{applied}}\;{\text{N}}\;{\text{of}}\;{\text{fertilizer}}\;{\text{in }}\;{\text{the }}\;{\text{treatment}}}}\times100$$

$${\text{NAE }}\left( {{\text{kg Nkg}}^{ - 1} } \right) = \frac{{{\text{Grain yield from the treatment}} - {\text{Grain yield from the control}}}}{{\text{Total applied N of fertilizer in the treatment}}}$$

$${\text{NPUE }}\left( {{\text{kg Nkg}}^{ - 1} } \right) = \frac{{\text{Total biomass}}}{{\text{Total N uptake}}}$$

## Supplementary information

Supplementary information 1

## Data Availability

Obtained with permission.
