# Supplementary material for: Effects of biochar-based controlled release nitrogen fertilizer on nitrogen-use efficiency of oilseed rape (Brassica napus L.)
Source: Sci Rep. 2020 Jul 6;10:11063. doi: 10.1038/s41598-020-67528-y (PMC7338421; doi:10.1038/s41598-020-67528-y)
Supplement: Supplementary file 1 — Supplementary information 1 [file 41598_2020_67528_MOESM1_ESM.docx]

**Supplementary**

**Effects of biochar-based controlled release nitrogen fertilizer on nitrogen-use efficiency of oilseed rape (Brassica napus L.)**

Jiayuan Liao^1,2#^, Xiangrong Liu^1,3#^, Ang Hu^1^, Haixing Song^1^, Xiuzhi Chen^2^, Zhenhua Zhang^1,4*^

^1^Southern Regional Collaborative Innovation Center for Grain and Oil Crops in China, College of Resources and Environmental Sciences, Hunan Agricultural University, Changsha 410128, China; ^2^Guangdong Province Key Laboratory for Climate Change and Natural Disaster Studies, School of Atmospheric Sciences, Sun Yat-sen University, Guangzhou 510275, China; ^3^Hengyang Branch of Hunan Tobacco Company, Hengyang, 421600, China; ^4^National Engineering Laboratory on Soil and Fertilizer Resources Efficient Utilization, Changsha 410128, Hunan Provincial Key Laboratory of Farmland Pollution Control and Agricultural Resources Use, Hunan Provincial Key Laboratory of Nutrition in Common University;

^#^These authors contributed equally to this work.

*e-mail: [zhzh1468@163.com](mailto:zhzh1468@163.com)

Table S1 Richness and alpha diversity of bacteria

| Treatment | ACE | Chao1 | Simpson | Shannon |
| --- | --- | --- | --- | --- |
| CK | 1017.0±9.64a | 1030.37±8.63a | 0.024±0.001a | 5.35±0.03a |
| B | 1039.7±4.20a | 1042.38±8.02a | 0.023±0.001ab | 5.33±0.04ab |
| BU | 1019.6±15.30a | 1026.58±15.74a | 0.022±0.001b | 5.14±0.11b |
| Urea | 1027.5±7.37a | 1038.31±10.99a | 0.021±0.003b | 5.10±0.12b |
| BCRNF | 1028.8±5.48a | 1035.70±10.41a | 0.022±0.002b | 5.20±0.09b |

Mean values (means ± SD, n=3) followed by different letters indicate significant difference between treat types at the p < 0.05 level. ACE, abundance-based coverage estimator.

Table S2 Soil functional gene abundance level under difference treatments

| Stage | Treatments | AOA | AOB | nirS | nirK | nosZ |
| --- | --- | --- | --- | --- | --- | --- |
|  |  | ×10^7^gene copies g^-1^ dry soil | | | | |
| Seedling stage | CK | 184.26±5.3a | 3.75±0.4d | 155.57±20.1a | 85.5±6.2c | 18.9±0.5a |
|  | B | 111.73±7.3a | 4.22±0.5d | 132.22±8.5ab | 23.45±6.5c | 20.75±1.5a |
|  | B+U | 104.48±0.6ab | 8.02±0.2b | 124.93±4.2b | 142.61±10.5a | 20.36±0.3a |
|  | Urea | 94.38±2.1b | 9.31±0.3a | 114.97±6.3b | 146.95±12.1a | 12.42±2.4b |
|  | BCRNF | 63.91±4.7c | 5.84±0.4c | 72.02±3.5c | 94.3±2.5b | 21.28±2.3a |
| Flowering stage | CK | 858.8±19.2a | 13.35±0.4c | 34.68±2.6c | 187.6±42.5b | 64.15±6.1b |
|  | B | 707.2±14.5b | 16.18±2.9c | 41.31±2.3c | 344.2±80.1a | 123.87±16.7a |
|  | B+U | 284.2±3.6c | 19.17±3.4bc | 150.41±7.2b | 37.65±5.1c | 49.47±9.8b |
|  | Urea | 173.7±3.7c | 24.61±2.9b | 285.12±31.5a | 166.67±16.5b | 71.54±8.5b |
|  | BCRNF | 173.8±2.6c | 50.05±1.2a | 207.15±3.8b | 24.34±6.0c | 127.85±23.5a |

CK: soil without nitrogen fertilizer; B: soil amended with biochar; B+U: soil amended with biochar and urea; Urea: soil amended with only urea as nitrogen fertilizer; BCRNF: soil amended with BCRNF as nitrogen fertilizer. Different small letters indicate significant differences between treatments (p < 0.05).

Table S3. Chemical properties of soils under different treatments

| Stage | Treatments | pH | SOC(g**﹒**kg^-1^) | TN(g**﹒**kg^-1^) | TP(g**﹒**kg^-1^) | TK(g**﹒**kg^-1^) | Olsen-P  (mg**﹒**kg^-1^) | Avail K  (mg**﹒**kg^-1^) |
| --- | --- | --- | --- | --- | --- | --- | --- | --- |
| Seedling stage | CK | 5.64±0.01a | 12.89±0.08b | 1.29±0.06bc | 0.23±0.03b | 4.39±0.19c | 12.34±0.44ab | 46.59±1.67a |
|  | B | 5.46±0.1a | 19.89±1.90a | 1.25±0.03c | 0.28±0.01a | 5.47±0.18a | 13.38±0.33ab | 33.39±1.93bc |
|  | B+U | 4.99±0.12b | 20.57±1.89a | 1.34±0.02ab | 0.29±0.02a | 5.15±0.38a | 13.64±0.71a | 40.02±1.81ab |
|  | Urea | 5.39±0.17a | 17.42±0.40b | 1.42±0.03a | 0.24±0.01b | 4.43±0.25b | 12.00±0.23b | 25.96±5.13c |
|  | BCRNF | 4.89±0.06b | 20.84±1.50a | 1.30±0.01bc | 0.30±0.01a | 5.07±0.24ab | 13.17±0.06ab | 31.04±1.80c |
| Harvest stage | CK | 5.70±0.07ab | 13.09±0.64b | 1.24±0.03b | 0.21±0.01b | 4.66±0.20b | 47.38±0.70b | 37.71±2.73a |
|  | B | 5.95±0.03a | 20.40±1.37a | 1.22±0.05b | 0.25±0.02a | 5.56±0.15a | 54.48±1.56a | 29.72±0.68b |
|  | B+U | 5.45±0.13bc | 21.98±1.42a | 1.23±0.02b | 0.27±0.02a | 4.96±0.13b | 49.72±2.03b | 29.93±1.02b |
|  | Urea | 5.58±0.12bc | 17.25±0.97b | 1.26±0.01b | 0.22±0.01b | 4.36±0.25b | 50.27±0.43b | 29.69±1.08b |
|  | BCRNF | 5.33±0.08c | 20.16±0.86a | 1.37±0.02a | 0.28±0.02a | 4.89±0.10b | 54.13±0.69a | 31.84±1.63b |

CK: soil without nitrogen fertilizer; B: soil amended with biochar; B+U: soil amended with biochar and urea; Urea: soil amended only with urea as nitrogen fertilizer; BCRNF: soil amended with BCRNF as nitrogen fertilizer. Different small letters indicate significant differences between different treatments (p < 0.05).

Table S4. Nutrient of plant under different treatments

| Stage | Treatments | TN concentration (mg·g^-1^) | TP concentration (mg·g^-1^) | TK concentration (mg·g^-1^) |
| --- | --- | --- | --- | --- |
| Seedling stage | CK | 35.70±1.216c | 2.22±0.09b | 12.63±1.03b |
|  | B | 38.12±0.51bc | 2.46±0.16b | 18.16±1.78a |
|  | B+U | 44.43±1.81a | 2.81±0.03a | 18.03±1.24a |
|  | Urea | 40.56±2.06ab | 2.48±0.04b | 15.95±1.44a |
|  | BCRNF | 43.71±3.91a | 2.94±0.09a | 18.16±1.78a |
| Bolting stage | CK | 28.14±0.18a | 2.14±0.14c | 12.28±1.79c |
|  | B | 30.53±0.37a | 2.62±0.05bc | 15.12±0.72b |
|  | B+U | 31.55±1.87a | 2.53±0.09ab | 15.54±1.42b |
|  | Urea | 26.33±2.08a | 2.54±0.06ab | 15.90±0.48ab |
|  | BCRNF | 31.76±0.82a | 2.29±0.11c | 17.37±2.44a |
| Flowering stage | CK | 22.78±0.46b | 1.39±0.05b | 8.88±0.12b |
|  | B | 24.11±1.35b | 1.73±0.06a | 9.85±0.42b |
|  | B+U | 30.38±1.94a | 1.65±0.06a | 8.89±0.81b |
|  | Urea | 29.28±0.74a | 1.71±0.04a | 11.00±1.06a |
|  | BCRNF | 28.71±2.25a | 1.66±0.03a | 10.94±0.63ab |
| Harvest stage | CK | 20.18±0.12b | 1.37±0.08c | 7.73±0.61b |
|  | B | 20.07±0.27b | 1.71±0.07b | 9.82±0.80a |
|  | B+U | 25.24±0.51a | 1.65±0.03b | 7.69±0.63b |
|  | Urea | 24.52±0.62a | 1.68±0.06b | 7.71±0.83b |
|  | BCRNF | 25.57±0.25a | 2.07±0.05a | 9.86±0.22a |

CK: soil without nitrogen fertilizer; B: soil amended with biochar; B+U: soil amended with biochar and urea; Urea: soil amended with only urea as nitrogen fertilizer; BCRNF: soil amended with BCRNF as nitrogen fertilizer. Different small letters indicate significant differences between treatments (p < 0.05).

Table S5. Physical and chemical properties of the selected soil.

| pH  value | SOC content  (g·kg^-1^) | TN content  (g·kg^-1^) | TP  content  (g·kg^-1^) | TK  content  (g·kg^-1^) | Olsen-P  content  (mg·kg^-1^) | AK  content  (mg·kg^-1^) | NO_3_^-^_-_N  content  (mg·kg^-1^) | NH_4_^+^-N  content  (mg·kg^-1^) |
| --- | --- | --- | --- | --- | --- | --- | --- | --- |
| 5.55 | 13.59 | 1.28 | 0.27 | 5.52 | 18.87 | 81.42 | 26.56 | 10.15 |

Table S6 Primers used for the real-time PCR

| Gene | Primer | Sequence | Reference |
| --- | --- | --- | --- |
| AOA^a^ | Arch-amoAF | STAATGGTCTGGCTTAGACG | 2 |
|  | Arch-amoAR | GCGGCCATCCATCTGTATGT |  |
| AOB^a^ | amoA-1F | GGGGTTTCTACTGGTGGT | 3 |
|  | amoA-2R | CCCCTCKGSAAAGCCTTCTTC |  |
| nirK | nirK876C | ATYGGCGGVCAYGGCGA | 4 |
|  | nirK1040 | GCCTCGATCAGRTTRTGG |  |
| nirS | cd3af | GTSAACGTSAAGGARACSGG | 5 |
|  | R3cd | GASTTCGGRTGSGTCTTGA |  |
| nosZ | nosZ-F | CGYTGTTCMTCGACAGCCAG | 6 |
|  | nosZ-R | CGSACCTTSTTGCCSTYGCG |  |

Note: a. AOA-ammonia-oxidizing archaea; AOB-ammonia-oxidizing bacteria.

**Biochar and BCRNF production.**

Biochar and BCRNF (13.2% N) were provided by the National Engineering Laboratory on Soil and Fertilizer Resources Efficient Utilization of the Hunan Agricultural University. Biochar were derived from oilseed rape straws collected from the ﬁeld station at Hunan Agricultural University, Southern China. Oilseed rape straws were air-dried, and then ground into particles with size smaller than 0.3 mm. Under a 3 L·min^-1^ N_2_ flow, straws were pyrolyzed at 400℃ for 3 h in a laboratory-scale pyrolysis unit comprising of a tube reactor equipped with a programmable temperature controller and a cooling system to collect condensed gas vapors and bio-oil^1^. All preparations were carried out in duplicate.

BCRNF were prepared via the incorporation of urea and bentonite into biochar through hydrothermal synthesis (Liu et al.2019). A solution containing 2 g of urea (^15^N labelled fertilizer), and 100 ml distilled water was introduced to a 250 ml sealed reactor containing 20 g of biochar at 135 ℃ for 2 hours (as precursor for BCRNF). When the reactor cooled down to room temperature, 2 g of bentonite and 1 g of PVA was mixed with the precursor. Then, the sealed reactor was transferred to an oven at 135 ℃ for 4 hours. The resulting product was dried to constant weight in an oven at 70 °C, and stored for future use, referred to as BCRNF. The total C, N, H and O, pH, ash content, surface area (SA) of biochar and BCRNF are summarized in Table S2, as reported by Liu et al.^1^. Urea particles filled the inner pores and channels of biochar and a new organic complex was generated. Scanning electron microscopy and gas adsorption were conducted to identify the urea-loading and storage of bentonite in the inner pores of the biochar particles. X-ray diffraction, Fourier transform infrared spectroscopic and X-ray photoelectron spectroscopic studies demonstrated that strengthening the interactions among biochar, urea, and bentonite, helps control the moisture diffusion and penetration of bentonite, thereby leading to nutrient retention, demonstrating favourable controlled-release properties of the BCRNF^1^.

Table S7. The physicochemical properties of samples.

| Samples | pH | Ash content (%) | Element analysis (%) | | | N (g/kg) | K (g/kg) | P  (g/kg) | Porosity parameters | | |
| --- | --- | --- | --- | --- | --- | --- | --- | --- | --- | --- | --- |
|  |  |  | C | H | O |  |  |  | SSA_BET_ (m^2^/g) | V_T_ (m^3^/g) | D_p_ (nm) |
| Biochar | 7.84 | 9.6 | 61.5 | 4.2 | 25.6 | 1.24 | 36.8 | 0.32 | 23.84 | 0.015 | 2.58 |
| BCRNF | 8.15 | 15.3 | 57.4 | 3.8 | 23.4 | 38.2 | 26.5 | 0.20 | 0.62 | 0.001 | 2.05 |


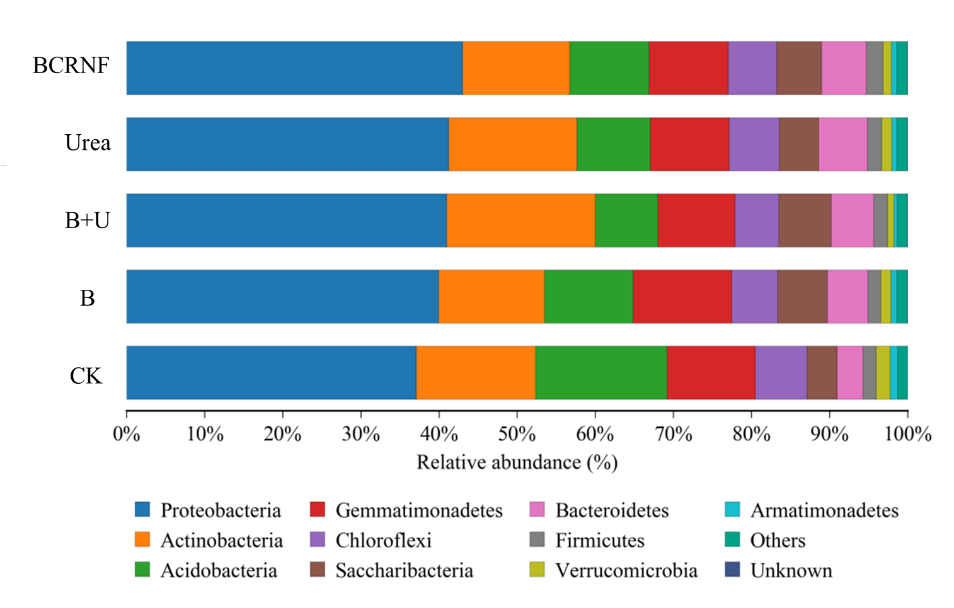


**Figure S1**. Relative abundances of bacterial community composition at phylum level under difference treatments. “Other” refers all other taxa with abundances lower than 0.9%. CK: untreated soil; B: soil treated with biochar; B+U: soil treated with biochar and urea; Urea: soil treated with only urea as nitrogen fertilizer; BCRNF: soil treated with BCRNF as nitrogen fertilizer.

**Figure S2.** Distribution of the top 30 abundant bacteria at the family level and their cluster analysis in different treatments as visualized by heatmaps (variables clustering on the vertical axis). The color intensity of the scale indicated the relative abundance of each family. CK: untreated soil; B: soil treated with biochar; B+U: soil treated with biochar and urea; Urea: soil treated with only urea as nitrogen fertilizer; BCRNF: soil treated with BCRNF as nitrogen fertilizer.

**Figure S3.** Effect of fertilizer treatments on Urease (a). FDA hydrolysis (b). Different small letters indicate significant difference between different treatments (p < 0.05).

**Figure S4.** Effect of fertilizer treatments on microbial biomass C (a). N (b). P(C). Different small letters indicate significant difference between different treatments (p < 0.05).

**Figure S5**. Effect of fertilizer treatments on potential nitrification rates. Different small letters indicate significant difference between different treatments (p < 0.05).

**Figure S6.** N release behavior of Urea, B+U and BCRNF in water.

**Figure S7**. Effect of fertilizer treatments on nitrogen physiological use efficiency (a), root activity (b), nitrate reductase (c), glutamine synthetase (d) of brassica napus. CK: soil without nitrogen fertilizer; B: soil amended with biochar; B+U: soil amended with biochar and urea; Urea: soil amended with only urea as nitrogen fertilizer; BCRNF: soil amended with BCRNF as nitrogen fertilizer. Different small letters indicate significant differences between different treatments (p <0.05).

**Figure S8**. N uptake and N distribution of plant with ^15^N tracer. Effect of fertilizer treatments on N uptake of B. napus (a). ^15^N distributions of grain in B. napus (b). N rate of grain and plant biomass on ^15^N derived from fertilizers (c). Different small letters indicate significant differences between different treatments (p <0.05).


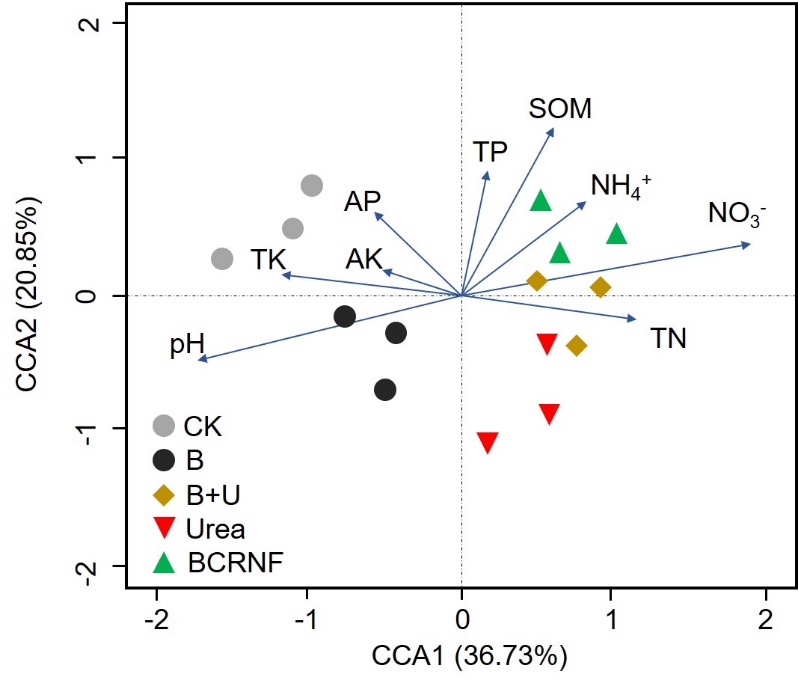


**Figure S9.** Canonical correlation analysis (CCA) of the effects of soil physiochemical properties on bacterial community composition among each treated soil. CCA ordination was plotted for the first two dominant dimensions representing the relationship between the bacterial community (symbols) and environmental factors (arrows).

**References**

1. Liu, X., Liao, J., Song, H., Yang, Y., Guan, C., Zhang, Z. A Biochar-Based Route for Environmentally Friendly Controlled Release of Nitrogen: Urea-Loaded Biochar and Bentonite Composite. Scientific reports, 9(1), 1-12(2019).

2. Francis, C. A., Roberts, K. J., Beman, J. M., Santoro, A. E., Oakley, B. B. Ubiquity and diversity of ammonia-oxidizing archaea in water columns and sediments of the ocean. Proceedings of the National Academy of Sciences, 102(41), 14683-14688(2005).

3. Rotthauwe, J. H., Witzel, K. P., Liesack, W. The ammonia monooxygenase structural gene amoA as a functional marker: molecular fine-scale analysis of natural ammonia-oxidizing populations. Appl. Environ. Microbiol., 63(12), 4704-4712(1997).

4. Henry, S., Baudoin, E., López-Gutiérrez, J. C., Martin-Laurent, F., Brauman, A., Philippot, L. Quantification of denitrifying bacteria in soils by nirK gene targeted real-time PCR. Journal of microbiological methods, 59(3), 327-335 (2004).

5. Throbäck, I. N., Enwall, K., Jarvis, Å., Hallin, S. Reassessing PCR primers targeting nirS, nirK and nosZ genes for community surveys of denitrifying bacteria with DGGE. FEMS microbiology ecology, 49(3), 401-417(2004).

6. Kloos, K., Mergel, A., Rösch, C., Bothe, H. Denitrification within the genus Azospirillum and other associative bacteria. Functional Plant Biology, 28(9), 991-998(2001).
